# BET and BRAF inhibitors act synergistically against *BRAF‐*mutant melanoma

**DOI:** 10.1002/cam4.667

**Published:** 2016-05-11

**Authors:** Luca Paoluzzi, Douglas Hanniford, Elena Sokolova, Iman Osman, Farbod Darvishian, Jinhua Wang, James E. Bradner, Eva Hernando

**Affiliations:** ^1^ New York University Cancer InstituteNew York University Langone Medical CenterNew YorkNew York; ^2^ Department of PathologyNew York University School of MedicineNew YorkNew York; ^3^ Interdisciplinary Melanoma Cooperative GroupNYU Cancer InstituteNYU Langone Medical CenterNew YorkNew York; ^4^ Department of DermatologyNew York University School of MedicineNew YorkNew York; ^5^ NYU Center for Health Informatics and BioinformaticsNew YorkNew York; ^6^ Dana Farber Cancer Institute, Harvard Medical SchoolBostonMassachusetts

**Keywords:** BET inhibition, *BRAF* inhibition, JQ1, Melanoma, Vemurafenib

## Abstract

Despite major advances in the treatment of metastatic melanoma, treatment failure is still inevitable in most cases. Manipulation of key epigenetic regulators, including inhibition of Bromodomain and extra‐terminal domain (BET) family members impairs cell proliferation in vitro and tumor growth in vivo in different cancers, including melanoma. Here, we investigated the effect of combining the BET inhibitor JQ1 with the BRAF inhibitor Vemurafenib in in vitro and in vivo models of *BRAF‐*mutant melanoma. We performed cytotoxicity and apoptosis assays, and a xenograft mouse model to determine the in vitro and in vivo efficacy of JQ1 in combination with Vemurafenib against *BRAF‐*mutant melanoma cell lines. Further, to investigate the molecular mechanisms underlying the effects of combined treatment, we conducted antibody arrays of in vitro drug‐treated cell lines and RNA sequencing of drug‐treated xenograft tumors. The combination of JQ1 and Vemurafenib acted synergistically in *BRAF*‐mutant cell lines, resulting in marked apoptosis in vitro*,* with upregulation of proapoptotic proteins. In vivo, combination treatment suppressed tumor growth and significantly improved survival compared to either drug alone. RNA sequencing of tumor tissues revealed almost four thousand genes that were uniquely modulated by the combination, with several anti‐apoptotic genes significantly down‐regulated. Collectively, our data provide a rationale for combined BET and BRAF inhibition as a novel strategy for the treatment of melanoma.

## Introduction

Treatment of metastatic melanoma remains a major clinical challenge, despite remarkable advances and novel approved compounds 1. Melanoma cells are exquisitely dependent on hyper‐activation of the MAPK‐signaling pathway, with activating mutations in *BRAF* (around 50%) or other pathway members as key drivers of tumorigenesis 2.

Since 2011, the FDA has approved three drugs that target the MAPK pathway and prolong overall and/or progression‐free survival: the BRAF inhibitors Vemurafenib and Dabrafenib and the MEK inhibitor Trametinib. Inhibition of this pathway has been a particularly effective strategy in melanoma, however, virtually all treated patients relapse after a relatively short time 3, 4. New treatment strategies to potentially prevent or overcome the emergence of drug resistance include the combination of inhibitors of the MAPK pathway with immunotherapies or with inhibitors of other aberrant cell signaling pathways common to melanoma 1.

Epigenetic dysregulation in melanoma is an emerging field of research. Our laboratory and others have recently elucidated a role for epigenetic regulators and histone variants in the pathogenesis of melanoma 5, 6 and demonstrated a critical role for the bromodomain (BrD)‐containing protein BRD4 in melanoma maintenance 7. BRD4 belongs to the BrD and extraterminal domain (BET) family of epigenetic “readers”, that bind to acetylated lysine residues of histones, to which they recruit chromatin‐modifying enzymes to effect transcriptional changes 8. BRD4 has been shown to exert oncogenic or tumor suppressor functions in various tumor types 9, 10, 11.

Recently, small molecule inhibitors have been developed that displace BRD‐containing proteins from chromatin. In particular, JQ1 is a small molecule that binds competitively to bromodomains with high potency for BRD4, and selectivity for BET proteins 12, 13. JQ1 and similar BET inhibitors are remarkably effective anti‐proliferative agents in vitro and in vivo for various cancers, including melanoma 14, 15, 16. In our previous study, we found that treatment with the BET inhibitor MS417 impaired melanoma cell proliferation in vitro and tumor growth and metastatic behavior in vivo, effects that were mostly recapitulated by BRD4 silencing 7. While BET inhibition alone has generally been more cytostatic than cytotoxic in preclinical models, combinations with other compounds have profoundly increased its anti‐neoplastic activity. For example, De Raedt et al. 17. recently demonstrated synergistic activity of JQ1 with the MEK inhibitor PD‐0325901 in in vitro and in vivo models of soft tissue sarcoma, with enhanced suppression of the Ras transcriptional output due to displacement of BRD4 from the promoters of repressed gene targets.

The rationale for combining BET and BRAF inhibitors in melanoma revolves around the hypothesis that both might trigger cell cycle arrest and apoptosis through different mechanisms of action. In this study, we assessed the effect of combining the BRAF inhibitor Vemurafenib with the BET inhibitor JQ1 in in vitro and in vivo models of *BRAF*‐mutant melanoma. We found that the two drugs interact synergistically in vitro*,* inducing significantly more apoptosis than either single drug. In a xenograft mouse model of *BRAF‐*mutant melanoma, combination therapy profoundly impaired tumor growth and prolonged animal survival compared to single agent groups.

## Materials and Methods

### Cell lines

A375, 451Lu, and SK‐MEL‐28 melanoma cell lines were acquired from the American Type Culture Collection. SK‐MEL‐100 was kindly provided by Dr. Alan Houghton (Memorial Sloan‐Kettering Cancer Center, New York, NY). All cells were cultured as previously described 7.

### Cytotoxicity assays

For all in vitro assays, cells were processed as previously described 7. JQ1, Vemurafenib and Trametinib were diluted in Dimethyl Sulfoxide (DMSO) maintained at a final concentration of <0.5%. For combination experiments, the final concentrations of each drug were selected to approximate the IC10‐50 for each drug. Cell‐Titer‐Glo Reagent (Promega Corporation, Madison, WI) and a SpectraMax M5 microplate reader (Molecular Devices, Sunnyvale, CA) were used as described previously 18. Each experiment was performed at least in duplicate and repeated at least twice. Data are presented as mean +/−SD.

### Flow cytometry

Cells were seeded at a density of 1 × 10^5^/mL and incubated with JQ1 with or without Vemurafenib at concentrations approximating the IC10‐30 for 48 h. For detection of apoptosis, Yo‐Pro‐1 and propidium iodide were used (Invitrogen, Grand Island, NY) as previously described 18. All data were analyzed with the Flowjo software (Tree Star, Ashland, OR). Each experiment was done at least in duplicate and repeated at least twice. The pancaspase inhibitor Q‐VD‐OPH hydrate (Q‐VD, Sigma‐Aldrich, St. Louis, MO) was used to test for caspase dependency. Data are presented as mean +/−SD.

### Cell‐cycle analysis

Cells were seeded at 1 × 10^5^ cells per well on 6‐well plates in triplicates. Drug concentrations that induced synergism and apoptosis were chosen for cell cycle analysis. The day after seeding, cells were treated with DMSO or Vemurafenib or JQ‐1 or their combination for 12 h cells, and suspended in 1:1000 of Vybrant DyeCycle Violet stain (Invitrogen). Cell cycle profiles were obtained with the Flowjo software.

### Apoptosis antibody array

Cell lysates from A375 and 451Lu were prepared after treatment with DMSO (control groups), JQ1, Vemurafenib or the combination for 48 h; lysates were analyzed using a human apoptosis array (RayBiotech, Norcross, GA) according to the manufacturer's instructions.

Signal intensities were quantified by densitometry using the software Image Studio Lite (Li‐Cor Biosciences, Lincoln, NE). After normalizing all groups to positive controls and to the DMSO treated group, groups were compared with significance defined by ratio fold changes <0.65 for protein down‐regulation and >2 for upregulation.

### Quantitative real‐time PCR

Total RNA was extracted using RNeasy Qiagen extraction kit according to the manufacturer's instructions. Five hundred nanograms of RNA were then subjected to DNase treatment and retro‐transcription. Real‐time PCR of the genes *TFPI2*,* AURKA*, and *PTTG1* was conducted using SYBR green fluorescence (Applied Biosystems Foster City, CA, USA). *GAPDH* and *GUSB* were used as internal standards. Relative quantification of gene expression was conducted with the 2^−∆∆t^ method 19.

### Mouse xenograft model

A375 melanoma cells were injected (1.5 × 10^6^/mouse) on both flanks of NOD/Scid/IL2*γ*R^−/−^ mice (NOG; *n *=* *40). Once tumors were palpable (volume around 100 mm^3^), mice were randomized in four groups of ten mice each and treated intraperitoneally (IP) with vehicle (5% DMSO + 10% 2‐hydroxypropyl‐*β*‐cyclodextrin) or JQ1 daily at 50 mg/kg JQ‐1 or Vemurafenib twice a day at 25 mg/kg for 8 days. Tumors were measured with standard calipers at least twice per week. Tumor volume was calculated using the formula 4/3*r* ^3^, where *r *= (*length* + *width*)/4. Animals were sacrificed when the tumor volume exceeded 2000 mm^3^ in accordance with institutional guidelines. On day+5, two mice per group were sacrificed in order to perform RNA sequencing on the removed tumors.

### Immunohistochemistry

Immunohistochemistry was performed on formalin fixed, paraffin‐embedded tissue sections as previously described 7. Antibodies used were rabbit anti‐mouse/human Ki67 clone SP6 at 1:400 (Thermo Scientific, Freemont, CA), and goat anti‐mouse CD105/Endoglin (R&D Systems Minneapolis, MN) at 1:200. For Ki67 evaluation, the area with the highest ki67 expression was identified on scanning magnification. The percentage of nuclei positive for ki67 was assessed in the latter area on high magnification (×400). TUNEL assay was performed to assess apoptosis.

### RNA sequencing

RNASequencing (RNAseq) libraries were prepared, using the mammalian RiboZero magnetic rRNA depletion kit (Epicenter, Madison, WI) following the manufacturer's protocol. Five nanograms of RiboZero‐treated RNA were used to construct a library, using Epicenter's ScriptSeq v2 RNASeq Library Preparation Kit. RNA sequencing reads were mapped to human genome hg19 with TopHat2 packages. The read counts table was analyzed with bioconductor package DEseq to get differential expression of each transcript with fold change, *P* value and False Discovery Rate (FDR) Genes with fold change above 2, *P* value<0.01 and FDR <0.1 were selected. Gene pathway analysis was done with gene set enrichment analysis (GSEA).

### Statistical analysis

Unless otherwise indicated, mean values ±SEM are representative of one of at least two independent experiments. Statistical significance was determined by unpaired *t* test (GraphPad Prism Software, La Jolla, CA). In the in vitro experiments, IC50 values for each cell line and drug–drug interactions in terms of synergy, additivity, or antagonism were computed as previously described (synergism was defined as a relative risk ratio less than one) 20.

In the mouse experiment, the log‐rank test was used to compare Kaplan–Meier Survival curves (GraphPad Prism Software).

## Results

### JQ1 interacts synergistically with Vemurafenib in *BRAF‐*mutant melanoma cell lines

**Figure 1:**
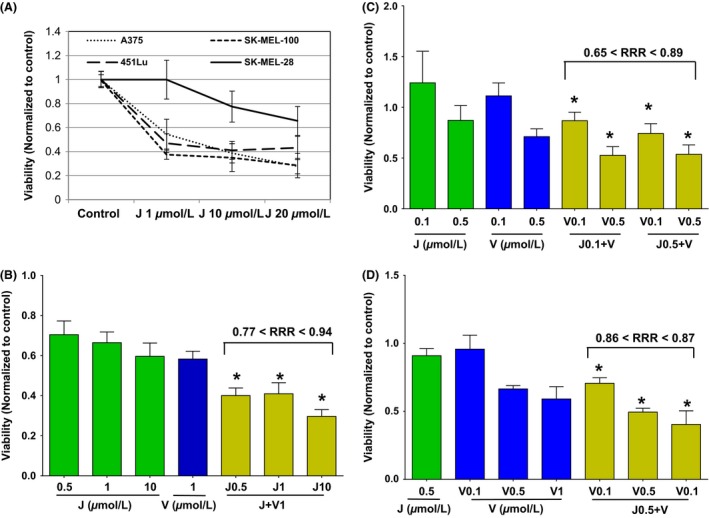
JQ1 (J) interacts synergistically with Vemurafenib (V) in *BRAF‐*mutant melanoma cell lines. (A) Percentage of viability relative to DMSO‐treated control of four melanoma cell lines after 72 h exposure to increasing combinations of JQ1. (B–D) Relative viability of 451Lu (B, *P ≤ 0.0005 vs. all groups), SK‐MEL‐28 (C, *P ≤ 0.3 vs. all groups) and A375 (D, *P ≤ 0.0001 vs. all groups) treated with various concentrations of J, V, or combinations. Ranges for average relative risk rations (RRR) are shown (RRR<1 defines synergism). Error bars represent mean +/−SD.

Similar to our previous work with different BET inhibitors in melanoma 7, the half maximal inhibitory concentration (IC50) values for JQ1 were variable across a panel of *BRAF‐*mutant melanoma cell lines ranging from less than 1 *μ*mol/L to more than 10 *μ*mol/L after 72 h of exposure (Fig. 1A; IC50 and confidence intervals (95% C.I.): SK‐MEL100 = 0.57 *μ*mol/L [0.1–2.1]; A375 = 3.08 *μ*mol/L [1.5–5.9]; 451Lu=1.09 *μ*mol/L [0.2–4.5]; SK‐MEL28 = 52 *μ*mol/L [44.5–61.9]). The duration of exposure to JQ1 appears to be a major determinant of cell viability, given that no significant changes were observed after shorter drug treatment (less than 72 h).

Combined treatment of JQ1 and Vemurafenib significantly decreased cell viability over either single treatment on three melanoma cell lines (Fig. 1B–D). These effects were synergistic as shown by relative risk ratio analysis (RRR<1) with RRRs less than 0.9 for 451Lu (*P* < 0.001 for the combination group versus all others), less than 0.8 for SK‐MEL‐28 (*P* ≤ 0.3), and equal to 0.8 for A375 (*P* < 0.001). These results demonstrate that BET and BRAF inhibitors synergistically suppress *BRAF*‐mutant melanoma. Adding the MEK inhibitor Trametinib to the combination of JQ1 and Vemurafenib resulted in significantly better cytotoxicity compared to all groups but the combination of Trametinib with JQ1 (*P* ≥ 0.28, Fig. S1).

### Combined JQ1 and Vemurafenib treatment of melanoma cell lines enhances apoptosis

Combination treatment of melanoma cells significantly reduced the percentage of cells in S‐phase after only 12 h of treatment (Fig. S2, *P* ≤ 0.001).

**Figure 2:**
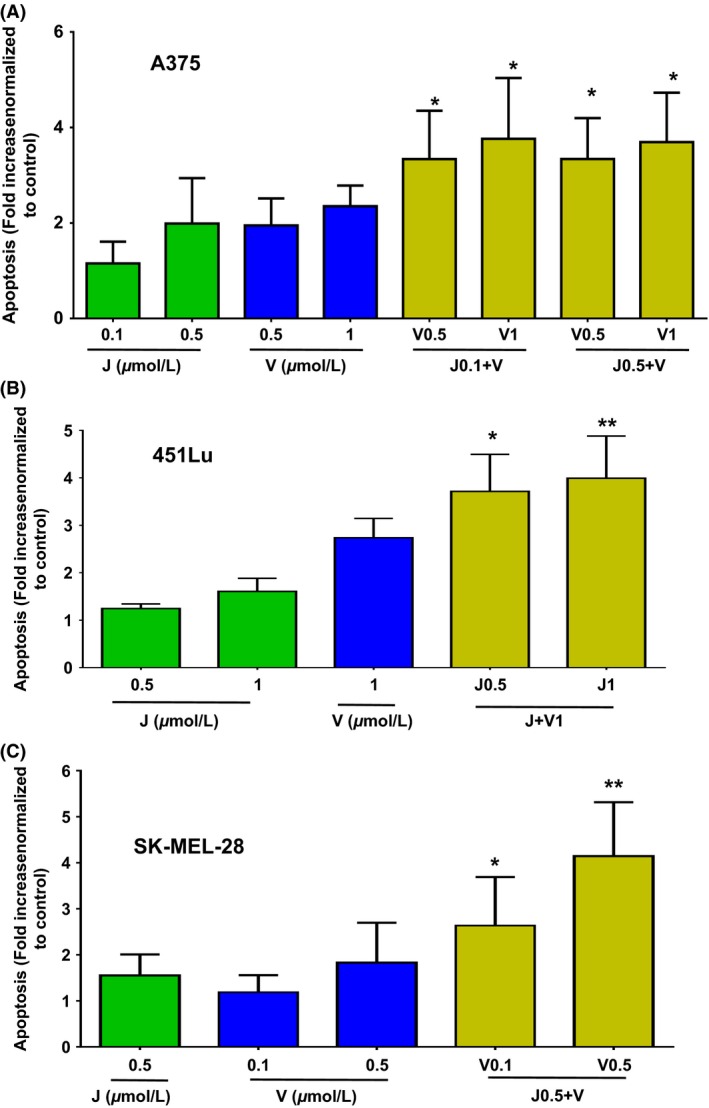
Enhanced apoptosis elicited by JQ1 (J) combined to Vemurafenib (V) in *BRAF‐*mutant melanoma cell lines. Fold increase in apoptotic cells relative to controls in A375 (A), 451Lu (B) and SK‐MEL‐28 (C). cells treated with various concentrations of J, V or combinations. Error bars represent mean +/−SD. *P < 0.05, **P < 0.01 for comparison of combination group to all single groups. Apoptosis in control group is approximately 10% for all cell lines.

JQ1 alone induced modest but statistically significant apoptosis versus vehicle control in three *BRAF*‐mutant cells lines (*P* = 0.010 for A375, *P* = 0.004 for 451Lu and *P* = 0.012 for SK‐MEL‐28, using JQ1 at 500 nmol/L). Combinations of JQ1 and Vemurafenib for 48 h triggered significantly more apoptosis than either compound alone in all three cell lines analyzed (*P* ≤ 0.014 for A375, Fig. 2A; *P* ≤ 0.015 for 451Lu, Fig. 2B; *P* ≤ 0.036 for SK‐MEL‐28, Fig. 2C). The use of the pan‐caspase inhibitor Q‐VD only partially decreased the rate of apoptosis in the A375 cell line (Fig. S3), suggesting that both, caspase‐dependent and independent mechanisms may be involved in triggering apoptosis. Collectively, these data demonstrate that concomitant BET and BRAF inhibition causes rapid growth arrest, followed by eventual induction of apoptosis.

### Combined JQ1 and Vemurafenib modulate the expression of apoptosis genes in vitro

**Figure 3:**
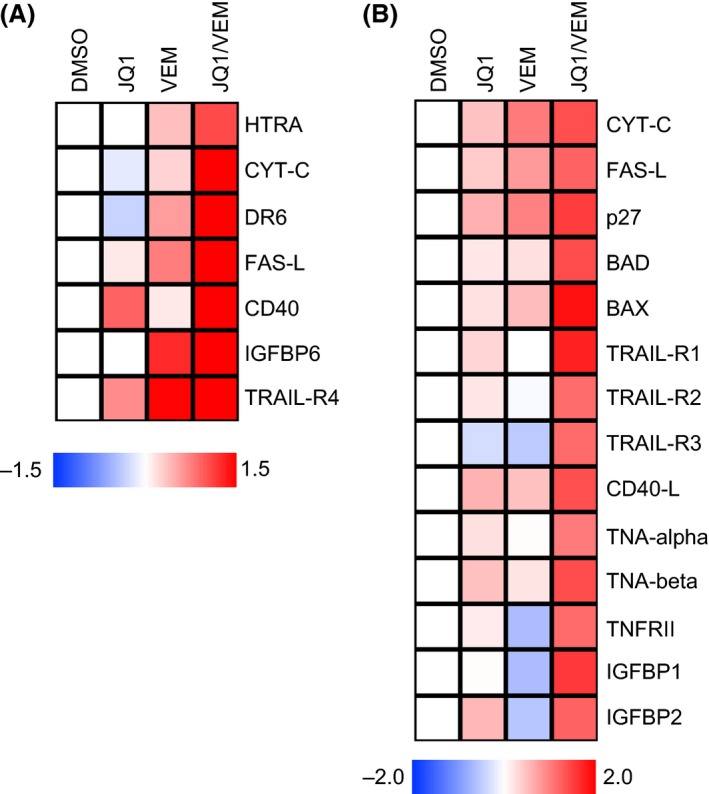
In vitro modulation of proteins involved in apoptosis after treatment with JQ1 alone or in combination with Vemurafenib. Protein lysates of 451Lu (A) and A375 (B) cells subjected to 48 h treatment with DMSO, JQ1 (500 nmol/L), Vemurafenib (500 nmol/L for A375, 1 *μ*mol/L for 451Lu) or their combination were analyzed by antibody arrays for apoptotic proteins expression. Heat maps depict of log2‐transformed normalized densitometry averages of experimental replicates for the most consistently altered proteins. Log2 ratios >1 or <−0.62 defines significant up or downregulation, as per manufacturer's recommendation. CD‐40L, CD40‐ligand; FASL‐L, FAS‐ligand; CYT‐C, cytochrome C; TRAIL‐R, TRAIL receptor; IGFBP, insulin growth factor‐binding protein.

We further investigated the increased apoptotic response elicited by the combined treatment in order to identify potential molecular mechanisms underlying this phenomenon. We analyzed protein lysates from two melanoma cell lines treated for 48 h with Vemurafenib, JQ1 or the combination, by antibody arrays containing 43 proteins involved in apoptosis and cell cycle (Fig. 3, Table S1).

Compared to all single treatment groups, we observed significant accumulation of pro‐apoptotic proteins in cells treated with the combination of JQ1 and Vemurafenib. In both cell lines tested, we observed a marked induction of FAS‐ligand, IGFBPs and TRAIL‐Receptors. Additionally, A375 displayed higher expression of BCL2 associate death promoter (BAD), BCL2 associated X protein (BAX), CD40‐ligand, TNF‐alpha and beta, STNFRII and p27; and 451Lu of HTRA, cytochrome C, DR6 and CD40, in response to the dual treatment.

In sum, combined treatment with BET and BRAF inhibitors elicited a robust apoptotic response through both the extrinsic or death receptor pathway (TRAIL‐R, FAS‐ligand) and the intrinsic or mitochondrial pathway (HTRA, BAX, BAD).

### JQ1 enhances the in vivo anti‐tumoral activity of Vemurafenib

**Figure 4:**
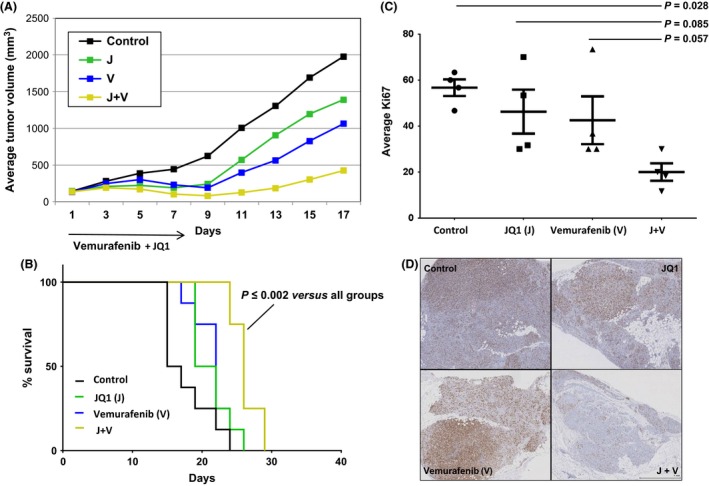
Enhanced activity of JQ1 (J) combined to Vemurafenib (V) in a xenograft NOG mouse model of *BRAF‐*mutant melanoma (A375). (A) Tumor growth curves of mice treated for 8 days with intraperitoneal J at 50 mg/kg per day, V at 25 mg/kg twice a day, or both. The combination resulted in reduced tumor growth compared to J, V and the control group since day+5 of treatment (*P* ≤ 0.01). (B) Kaplan–Meier survival curves. Mice in the combination group had significantly longer survival compared to all other groups starting from day+22 (*P* ≤ 0.03). (C) Relative Ki67 immunostaining levels on four tumors per group on day+5. (D) Representative micrographs of Ki67 immunostaining in each group.

We have previously shown the anti‐tumoral activity of BET inhibitors as single agents in melanoma in vivo 7. To examine possible synergy of BET and BRAF inhibitor combinations in vivo*,* we assessed single agent and combination therapy in a preclinical xenograft model of *BRAF*‐mutant melanoma. We administered JQ1 and Vemurafenib at doses associated with limited systemic toxicity when used separately in prior xenograft studies 12, 14, 21. Treatment with daily JQ1 combined with Vemurafenib for 8 days, was generally well‐tolerated with only 2 out of 8 mice in the combination group and 1 of 8 mice in the JQ1 group experiencing significant, transient weight loss (>10% of the initial weight, regained within a few days after discontinuation of treatment). Weight analysis on day +9 (day+8 was the last day of treatment) showed significant differences only between the combination and the control groups (*P* = 0.022, Table S2A). Beginning at day+5 from initiation of treatment, combined JQ1 and Vemurafenib treatment had statistically superior anti‐tumoral effect over JQ1 (*P* = 0.013) or Vemurafenib (*P* ≤ 0.0001) alone, or vehicle treatment (*P* ≤ 0.0001; Fig. 4A, Table S2B). One mouse in the combination group experienced a complete response by day+5 (no palpable tumor) though it subsequently relapsed by day+15 (treatment was stopped on day+8 because of significant weight loss in 2 out of 8 mice). No complete remissions were observed in the other groups in the studied timeframe. Time to endpoint analysis (the time at which tumor volume reached a certain maximum) analyzed by Kaplan–Meier and log‐rank test analyses revealed statistically significant longer survival for mice in the combined treatment group compared to all other groups (*P* ≤ 0.003, Fig. 4B, Table S2C).

Immunohistochemistry of tumors from mice euthanized on day+5 of treatment showed reduced proliferative index, as assessed by Ki67 staining, in the combined treatment group versus all other groups (Fig. 4C and D). TUNEL assay did not show significant apoptosis in any of the treated groups compared to the control (Fig. S4).

In sum, these results demonstrate that the combination of BRAF and BET inhibitors exert cooperative therapeutic effects against melanoma in vivo, resulting in significant tumor growth control and extended mice survival.

### Combined JQ1 and Vemurafenib treatment significantly impacts transcriptional programs that control cell cycle regulation in vivo

**Figure 5:**
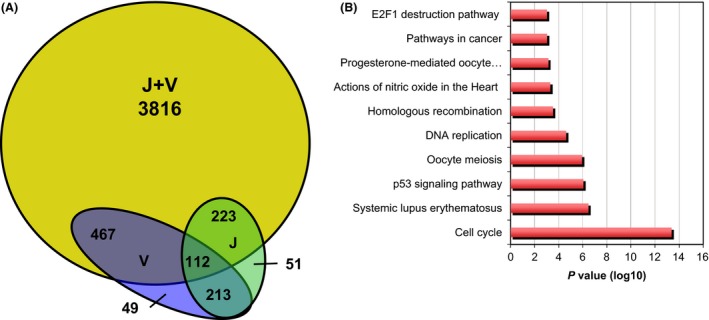
Combined JQ1 (J) and Vemurafenib (V) treatment significantly impacts transcriptional programs in vivo. (A) Venn diagram indicating the number of differentially expressed genes in tumors of each group treatment compared to control. J+V group shows a high number of genes (*n* = 3816) that are not shared with any of the other groups. (B) Gene set enrichment (GSE) analysis of genes significantly downregulated in J+V group compared to all others.

To investigate the molecular mechanisms controlling tumor growth effects of BRAF/BET inhibition, we performed RNA sequencing followed by gene set enrichment analysis (GSEA) of drug‐treated tumors. We assessed tumors from mice euthanized on day+5 (*n* = 4; 2 mice/group, 2 tumors/mouse) to identify the early impact of the combination of BRAF and BET inhibitors. The combination group showed a distinct and profound effect on the transcriptome compared to any singly treated tumors, with the largest number of genes differentially expressed (*n* = 3816 with *n* = 2201 significantly downregulated genes, Fig. 5A).

Interestingly numerous apoptotic regulators were uniquely downregulated by the combination (*n* = 114), including the anti‐apoptotic genes *BCL2, MCL1, BCL‐XL, BIRC5*, and *APAF1*. Other survival factors, such as *AKT1* and *TGFB1* were also significantly downregulated (Table S3A). These data support our in vitro finding that combined BET and BRAF inhibition suppresses cell proliferation and induces apoptosis.

In addition to the effects on cell cycle and apoptosis, almost thirty transcriptional regulators were significantly downregulated (such as *EZH2*) or upregulated (such as *EHF*) specifically in the combination group (Table S3B), suggesting combined BRAF and BET inhibition broadly shifts the transcriptional landscape of melanoma cells.

As for the gene sets specifically suppressed by the combined treatment, GSEA revealed cell cycle regulators (including *CDC25C,* and *E2F1*), p53‐related genes (such as *CCNE1*,* CHEK1*) and cancer‐related pathways (Fig. 5B and Table S4). Selected genes that were uniquely downregulated by the combined treatment were confirmed by quantitative PCR (Fig. S2).

Additional genes were significantly modulated by both, the combination and JQ1 or Vemurafenib alone, with most of them changing in the same direction (and the combination group significantly exacerbating the extent of modulation, Fig. S3). GSEA of these commonly regulated genes showed downregulation of genes related to cell cycle (such as *CCNB1, CDC25A*), DNA replication, CDK regulation of DNA replication, and base excision repair (Fig. S4). Of note, several genes related to angiogenesis such as *MMP1* and *TGFA* were significantly downregulated in combination‐treated tumors.

Collectively, these analyses support that the synergistic therapeutic potential of combined BRAF and BET inhibitors in melanoma results from distinct modulation of transcriptional programs controlling cell cycle, proliferation, and apoptosis.

## Discussion

The experiments presented here support the combination of the BET inhibitor JQ1 with the BRAF inhibitor Vemurafenib for the treatment of *BRAF*‐mutant melanoma patients. Our data suggest a synergistic interaction for the combination, both in vitro and in vivo. Mechanistically, combined treatment‐induced significantly more apoptosis than either drug alone in vitro and dramatically altered the transcriptional landscape of combination‐treated xenograft tumors in vivo.

How the combination with BRAF inhibitors exacerbates the transcriptional effects of BET inhibition remains uncertain. Recent studies have demonstrated that BRD4 binds preferentially to super enhancers (SEs), long stretches of enhancers that control the expression of ‘cell identity’ genes in normal cells and tumor‐specific oncogenes in cancer cells 22. Addiction to high‐level expression of these SE‐regulated oncogenes is thought to contribute to tumor cells vulnerability to BET inhibitors 23. The transcriptional output of BRD4 displacement and enhancer disruption may be modified by MAPK‐signaling inhibition, which is known to control numerous epigenetic regulators and transcription factors (i.e. P300**)** 24. In support of this crosstalk, recent studies have shown that loss of members of the Polycomb repressive complex PRC2 amplifies Ras‐driven transcription by triggering an epigenetic switch that sensitizes some tumors, including melanoma, to BRD4 inhibition 17.

In vitro, we found a striking upregulation of proapoptotic proteins in melanoma cell lines treated with combined BET and BRAF inhibitors, compared to any single‐agent treatment.

Treatment of melanoma cells with BET or BRAF inhibitors as single agents has been shown to induce apoptosis through the activation of the mitochondrial pathway 16, 23. Intriguingly, in our in vitro experiments, we observed increased expression of proteins involved in both the extrinsic and intrinsic pathways of apoptosis with modulation of key players such as TRAIL receptors, FAS‐ligand, cytochrome C, BAD, and BAX. Additional apoptotic regulators that were found induced by the combined treatment included CD40, CD40‐ligand, HTRA, or IGFBPs. Insulin‐like‐growth‐factor‐binding‐proteins have shown to contrast cancer progression by modulating the activity of insulin‐like growth factors 25. With regard to CD40 and its ligand, Pirozzi et al. 26. showed that a CD40‐dependent pathway is able to enhance T‐cell proliferation in vitro.

Overall these findings may help to explain the potent anti‐proliferative and pro‐apoptotic effect of the combination of BET and BRAF inhibitors. In particular, one explanation for their cooperative effects may rely on the ability of these drugs to induce apoptosis through multiple pathways when combined compared to their use as single agents.

In vivo, JQ1 and Vemurafenib combination was statistically significantly more efficacious than any other group from day+5 of treatment, which resulted in increased survival for mice in the combined treatment group compared to others. In order to investigate potential changes in cellular pathways that could underlie the in vivo therapeutic effects, we performed RNA sequencing of tumors obtained from mice sacrificed on day+5. Interestingly, tumors in the combination group showed a vast repertoire of uniquely modulated genes compared to all other groups (*n* = 3816). Genes significantly downregulated included cell cycle, DNA replication genes and regulators of DNA repair such as CHK. Inhibition of CHK1 through specific drug inhibitors has shown to drive melanoma cells into aberrant mitosis and apoptosis 27.

Additional downregulated genes were transcription factors such as *E2F1*,* E2F8* or *EHF*. E2F1 silencing has shown to associate with cell cycle arrest and significant reduction of Ki67 staining in an in vivo melanoma xenograft model 28. Of note, p53‐related genes coding for proteins actively involved in proliferation such as cyclins (*CCNE1‐2* for e.g.), were also found downregulated. Consistent with the anti‐proliferative signature revealed by transcriptomic profiling, immunohistochemistry of the same tumors revealed reduced proliferation in the combined treatment group (Ki67 staining).

In contrast to our in vivo findings, increased apoptosis prevailed over reduced cell proliferation in vitro. This discrepancy may be related to the timing of tumor collection, and/or differences in intracellular drug concentrations achieved in vitro and in vivo. Despite these differences, numerous apoptotic and pro‐survival genes were specifically downregulated in vivo upon combined BRAF and BET treatment, including the anti‐apoptotic proteins *BCL2, BCL‐XL*, and *MCL1,* and the survival factors *AKT1* and *HDAC1*. The BCL‐2 family of proteins has shown to influence both the progression and chemotherapeutic response of melanoma 29. Recent studies demonstrated that targeting this family of proteins with small molecule inhibitors such as ABT737 or TW‐37 can effectively trigger apoptosis through the activation of the mitochondrial pathway 29. Also, resistance to BRAF or MEK inhibitors is associated with the induction or persistence of AKT signaling in the presence of these drugs. Concomitant pharmacologic targeting of the MAPK and AKT pathways with BRAF and mTOR inhibitors is synergistic and reverses cross resistance of melanoma cells to BRAF and MEK inhibitors 30. With regard to HDAC1, Bandyopadhyay et al. 31 have shown that histone deacetylase 1 can affect histones but also other proteins, suppressing the transcriptional activity of deacetylated proteins such as p53. Consistently, HDAC1 silencing resulted in enhanced cytotoxicity in metastatic melanoma cells in vitro and in vivo 32. Hence, suppression of survival mechanisms, such as anti‐apoptotic factors, AKT signaling or HDAC activity could contribute to the enhanced anti‐tumoral effect of combined BET and BRAF inhibition. In sum, the improved anti‐tumor activity of the combination observed in vivo may rely on pleiotropic effects on multiple pathways including tumor cell proliferation and survival.

In conclusion, the combination of a BET inhibitor and a BRAF inhibitor has superior therapeutic potential than either drug alone in in vitro and in vivo models of *BRAF‐*mutant melanoma. Phase I and II clinical studies are currently exploring the safety and single agent activity of BET inhibitors in different solid tumors and hematologic malignancies 33, 34.

At the time our experiments were planned and finalized, the BRAF inhibitor Vemurafenib was the only FDA approved targeted treatment for patients with metastatic melanoma harboring the *BRAF* mutation. Recent phase III clinical trials have shown a prolongation in progression‐free survival and overall survival with the combination of a BRAF with an MEK inhibitor 4, 35.

We believe that the combination of BET inhibitors with a BRAF and/or an MEK inhibitor is a promising new strategy that warrants further preclinical and clinical development against *BRAF*‐mutant melanoma tumors.

## Conflicts of Interest

J. Bradner is consultant with stock ownership for Tensha Therapeutics, Acetylon Pharmaceuticals, Shape Pharmaceuticals, Syros Pharmaceuticals, and Agios Pharmaceuticals. All other authors have no potential conflicts of interest.

## Supporting information


**Figure S1.** The combination of JQ1 (J), Vemurafenib (V) and Trametinib (T) is not better than all groups.
**Figure S2.** Treatment of two cell lines with JQ1 (J) + Vemurafenib (V) affects cell cycle in vitro.
**Figure S3.** Apoptosis induced by JQ1 plus Vemurafenib (Vem) is partially caspase dependent.
**Figure S4.** Combination of JQ1 (J) plus Vemurafenib (V) does not significantly affect apoptosis in the xenograft melanoma model (A375).
**Figure S5.** Reduced expression of selected proliferation genes in the xenograft melanoma model (A375).
**Figure S6.** Combined JQ1 (J) plus Vemurafenib (V) treatment significantly impacts transcriptional programs *in vivo*.
**Figure S7.** GSE analysis of genes commonly modulated by JQ1 + Vemurafenib and Vemurafenib alone in tumors resected from mice on day + 5 of treatment.
**Table S1.** In vitro modulation of proteins involved in apoptosis after treatment with JQ‐1 plus/minus Vemurafenib.
**Table S2.** Combination of JQ1 (J) plus Vemurafenib (V) significantly affects tumor volume and survival in the xenograft melanoma model (A375).
**Table S3.** Modulation of apoptotic genes and transcription factors upon treatment with Vemurafenib and JQ1 in a xenograft melanoma model (A375).
**Table S4.** Selected examples of genes found downregulated in the JQ1 (J) + Vemurafenib (V) group versus all other groups in the gene set enrichment analysis from RNA sequencing of resected tumors.
