# Supplementary material for: BET and BRAF inhibitors act synergistically against BRAF‐mutant melanoma
Source: Cancer Med. 2016 May 11;5(6):1183–93. doi: 10.1002/cam4.667 (PMC4867668; doi:10.1002/cam4.667)
Supplement: Supplementary file 2 [file CAM4-5-1183-s002.docx]

**Supplementary Table 1:** **In vitro modulation of proteins involved in apoptosis after treatment with JQ-1 plus/minus Vemurafenib.** Two independent experiments in duplicate were performed for each cell line after treatment for 48h with DMSO (control), JQ1 (500nM), Vemurafenib (500nM for A375, 1uM for 451Lu) or their combination. Ratios of averages are shown after normalization to each correspondent control group for more differentially expressed proteins. Ratio > 2 defines significant overexpression (in bold), < 0.65 down-regulation, as per manufacturer.

**Supplementary Table 2. Combination of JQ1 (J) plus Vemurafenib (V) significantly affects tumor volume and survival in the xenograft melanoma model (A375).** (a) Weight analysis on day +9 (day+8 was last day of treatment) with comparison among all groups. (b) Tumor volumes with standard deviations for all groups at each time point are shown. (c) Survival analysis with all comparisons related to the last day of experiment (day+29). All significance testing was done at the p<0.05 level. Tx=treatment

**Supplementary Table 3. Modulation of apoptotic genes and transcription factors upon treatment with Vemurafenib and JQ1 in a xenograft melanoma model (A375).** Examples of apoptotic genes (a) and transcriptional regulators (b) from the RNA sequencing data that are significantly down- or up-regulated in JQ1 + Vemurafenib group compared to all other groups are shown.

**Supplementary Table 4.** Selected examples of genes found down-regulated in the JQ1 (J) + Vemurafenib (V) group versus all other groups in the gene set enrichment analysis from RNA sequencing of resected tumors.

**Supplementary Figure 1. The combination of JQ1 (J), Vemurafenib (V) and Trametinib (T) is not better than all groups.** A375 cells were treated for 48h with either JQ1 at 0.5uM, 1uM or 5uM plus or minus Vemurafenib at 0.5uM plus or minus Trametinib at 0.1uM or 0.5uM. Comparisons among all groups with correspondent p values after detection of a cytoxicity assay are shown. NS=not significant.

**Supplementary Figure 2. Treatment of two cell lines with JQ1 (J) + Vemurafenib (V) affects cell cycle in vitro.** Cell cycle analysis after 12h of exposure to J at 500nM, V at 1uM or both in A375 and 451Lu cells. Significant decrease in S-phase fraction is noted in both cell lines. *p=0.01-0.05, **p=0.001-0.01, ***p<0.001. Error bars represent mean +/- SD.

**Supplementary Figure 3. Apoptosis induced by JQ1 plus Vemurafenib (Vem) is partially caspase dependent.** A375 cells were treated for 48h with either JQ1 at 500nM, Vemurafenib at 1uM or the combination. Percentages of apoptotic cells are normalized to controls. Error bars represent mean +/- SD.

**Supplementary Figure 4. Combination of JQ1 (J) plus Vemurafenib (V) does not significantly affect apoptosis in the xenograft melanoma model (A375).** Tunel assay was performed on tumors resected on day+5. Purple color represent hematoxylin staining (darker staining for J+V group is related to sample processing). No significant apoptosis (brown color) is noted through all groups.

**Supplementary Figure 5. Reduced expression of selected proliferation genes in the xenograft melanoma model (A375).** RT-PCR of genes selected for validation of RNA sequencing data from A375 tumors treated with JQ1 (J), Vemurafenib (V) or the combination in the *in vivo* experiment. Significant less expression of *PTTG1* (A), *TFPI2* (B) and *AURKA* (C) genes is noted in the J+V group compared to other groups. Four tumors in each group with three replicates for each tumor were analyzed. *p=0.01-0.05, **p=0.057

**Supplementary Figure 6: Combined JQ1 (J) plus Vemurafenib (V) treatment significantly impacts transcriptional programs *in vivo*.** Fold change for modulated genes shared by V and J+V treated tumors (a) or J and J+V treated tumors (b) versus control.

**Supplementary Figure 7: GSE analysis of genes commonly modulated by JQ1+Vemurafenib and Vemurafenib alone in tumors resected from mice on day+5 of treatment.** Genes included in each category are significantly down-regulated by both treatments (J alone and J+V, or V alone and J+V) with the combined treatment significantly exacerbating the extent of modulation compared to Vemurafenib alone. Tumors were resected from mice on day+5 of treatment.
